# Supplementary figures and images for: Treatment outcomes in people with diabetes and multidrug-resistant tuberculosis (MDR TB) enrolled in the STREAM clinical trial
Source: PLOS Glob Public Health. 2025 Apr 1;5(4):e0004259. doi: 10.1371/journal.pgph.0004259 (PMC11960897; doi:10.1371/journal.pgph.0004259)

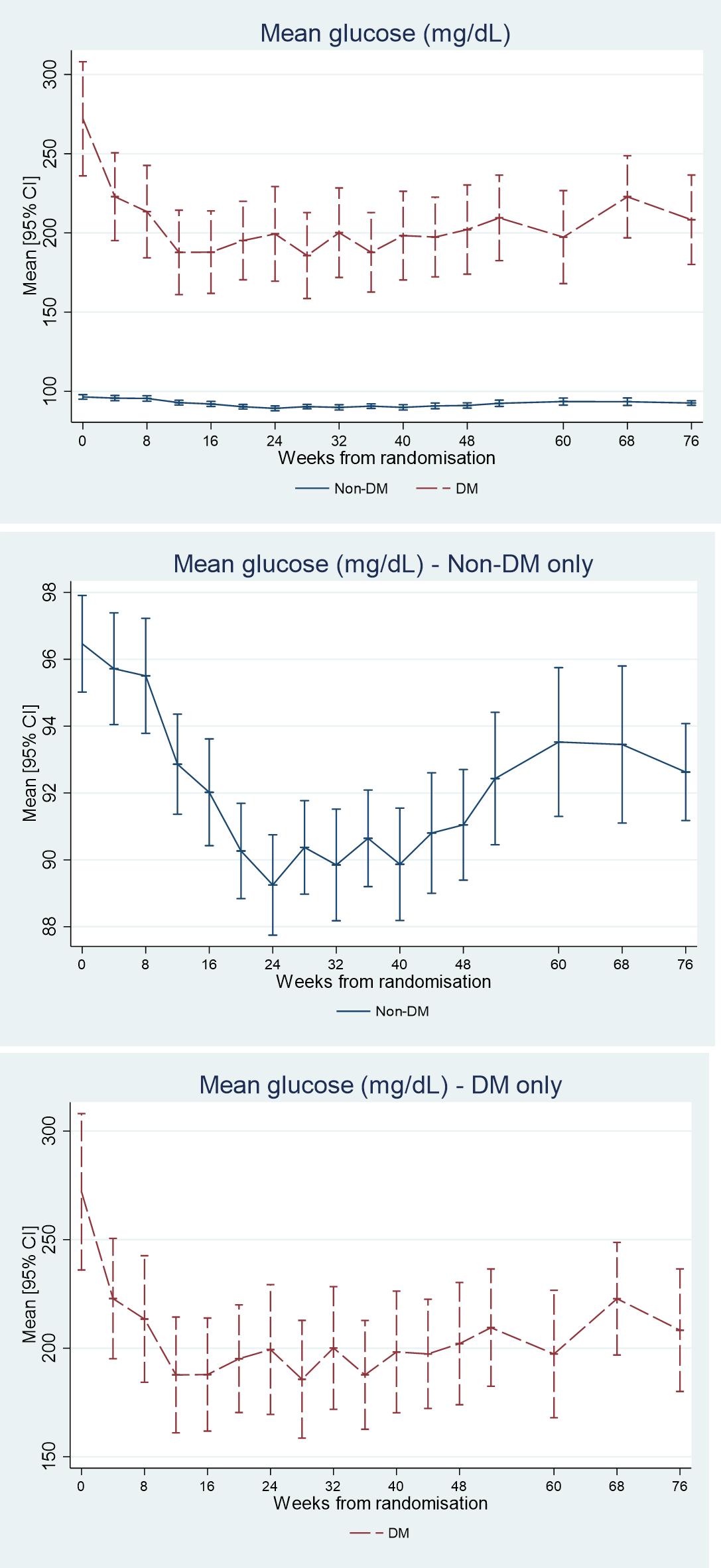

Supplement: S1 Fig — (TIF) [file pgph.0004259.s001.tif]

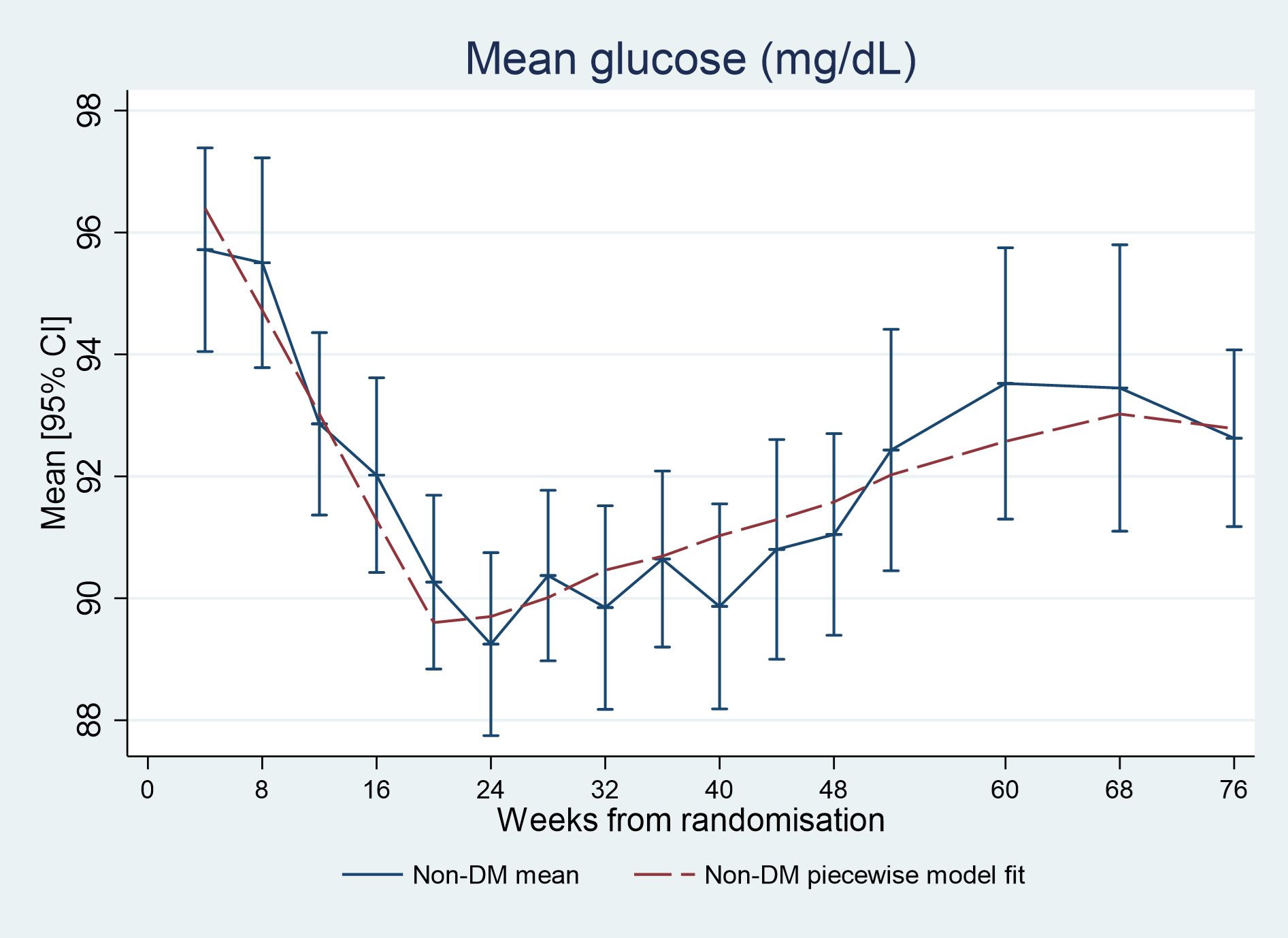

Supplement: S2 Fig — (TIF) [file pgph.0004259.s004.tif]

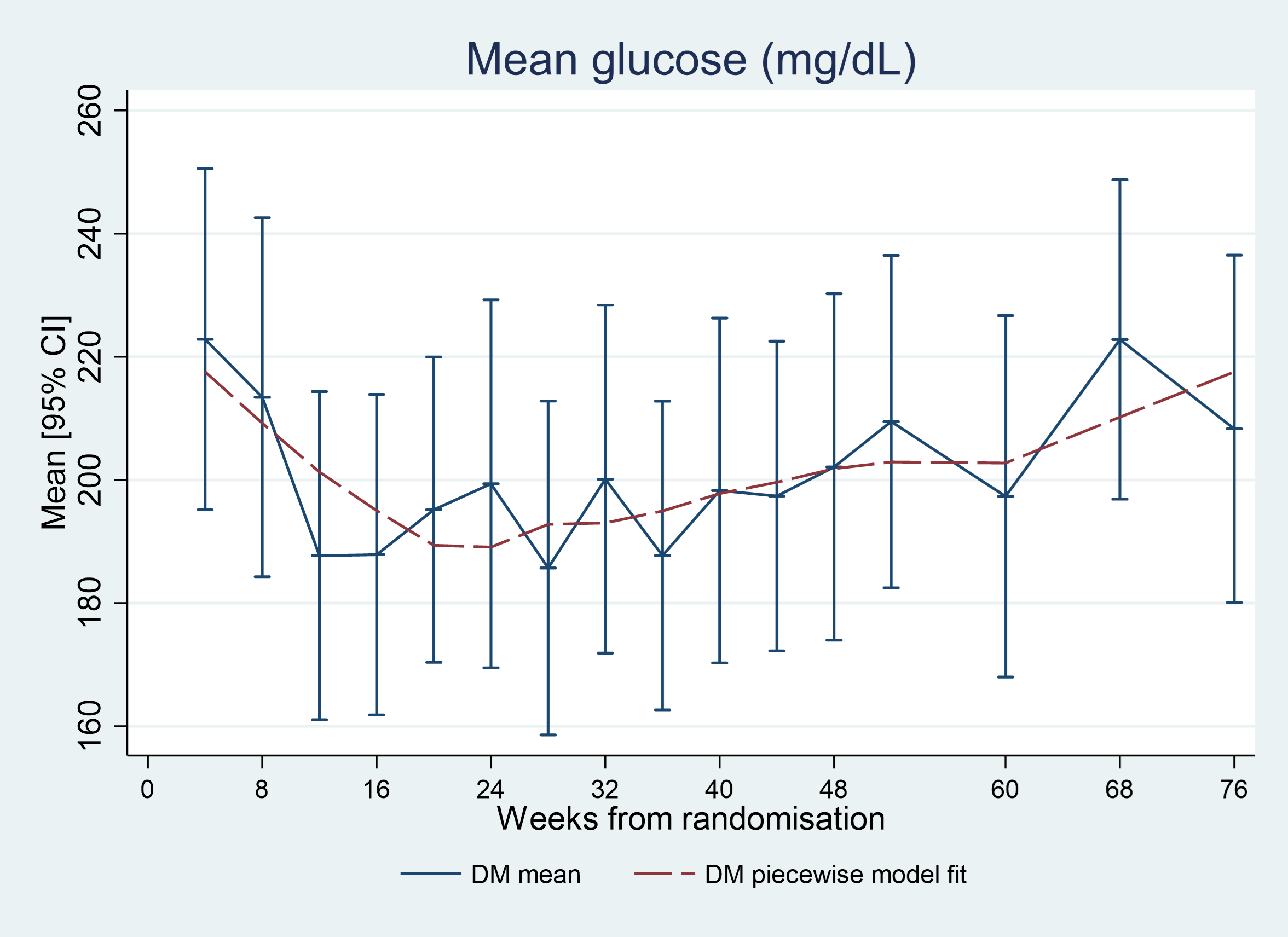

Supplement: S3 Fig — (TIF) [file pgph.0004259.s006.tif]
