# Supplementary material for: Treatment outcomes in people with diabetes and multidrug-resistant tuberculosis (MDR TB) enrolled in the STREAM clinical trial
Source: PLOS Glob Public Health. 2025 Apr 1;5(4):e0004259. doi: 10.1371/journal.pgph.0004259 (PMC11960897; doi:10.1371/journal.pgph.0004259)
Supplement: S1 Table — (DOCX) [file pgph.0004259.s002.docx]

**S1 Table** Baseline characteristics of glycemic control analysis population.

|  | **Non-DM**  **N=490** | **DM**  **N=49** | **P-value** |
| --- | --- | --- | --- |
| *Country, no. (%)* |  |  |  |
| Ethiopia | 61 (12) | 0 | <0.001 |
| Georgia | 30 (6) | 2 (4) |  |
| India | 99 (20) | 39 (80) |  |
| Moldova | 59 (12) | 0 |  |
| Mongolia | 114 (23) | 8 (16) |  |
| South Africa | 73 (15) | 0 |  |
| Uganda | 54 (11) | 0 |  |
| *Male, no. (%)* | 295 (60) | 36 (73) | 0.069 |
| *Age (years), no. (%)* |  |  |  |
| 15 – 24 | 114 (23) | 0 | <0.001 |
| 25 – 34 | 192 (39) | 5 (10) |  |
| 35 – 44 | 102 (21) | 18 (37) |  |
| 45 + | 82 (17) | 26 (53) |  |
| *BMI Category (kg/m^2^), no. (%)* |  |  |  |
| Severely underweight (< 16) | 72 (15) | 1 (2) | <0.001 |
| Underweight (16 – 18.49) | 143 (29) | 7 (14) |  |
| Normal (18.5 – 24.99) | 242 (49) | 32 (65) |  |
| Overweight (> 25) | 33 (7) | 9 (18) |  |
| *HIV+, no. (%)* | 79 (16) | 0 | <0.001 |
| *Smoking status, no. (%)* |  |  |  |
| Never smoked | 307 (63) | 36 (73) | 0.053 |
| Current smoker | 113 (23) | 4 (8) |  |
| Ex-smoker | 70 (14) | 9 (18) |  |
| *Extent of opacities, no./total (%)* |  |  |  |
| Minimal | 46/459 (10) | 3/46 (7) | 0.262 |
| Moderate | 252/459 (55) | 31/46 (67) |  |
| Advanced | 161/459 (35) | 12/46 (26) |  |
| *Number of cavities, no./total (%)* |  |  |  |
| None | 117/459 (25) | 9/46 (20) | 0.042 |
| Single | 80/459 (17) | 15/46 (33) |  |
| Multiple | 262/459 (57) | 22/46 (48) |  |
