# Supplementary material for: Treatment outcomes in people with diabetes and multidrug-resistant tuberculosis (MDR TB) enrolled in the STREAM clinical trial
Source: PLOS Glob Public Health. 2025 Apr 1;5(4):e0004259. doi: 10.1371/journal.pgph.0004259 (PMC11960897; doi:10.1371/journal.pgph.0004259)
Supplement: S2 Table — (DOCX) [file pgph.0004259.s003.docx]

**S2 Table** Final piecewise longitudinal model for glucose levels (non-DM group)

|  | **Parameter estimate (95% CI)** | **P-value** | **P-value for interaction** |
| --- | --- | --- | --- |
| *Intercept* | 87.68 (81.86, 93.49) | <0.001 | # |
| *Baseline glucose* | 0.14 (0.08, 0.20) | <0.001 | # |
| *Time (Up to 20 weeks)* | -0.42 (-0.51, -0.33) | <0.001 | # |
| *Time (Slope difference from 20-76 weeks)* | 0.49 (0.38, 0.60) | <0.001 | # |
| *Treatment regimen* |  |  | 0.592 |
| Long | 1.26 (-2.99, 5.52) | 0.562 |  |
| Short | Reference | - |  |
| Oral | -2.09 (-4.19, 0.00) | 0.051 |  |
| Six-month | -4.36 (-6.70, -2.03) | <0.001 |  |
| *Female* | -0.73 (-2.64, 1.19) | 0.457 | 0.094 |
| *Age (years)* |  |  | 0.042 |
| 15 – 24 | -1.96 (-4.32, 0.40) | 0.104 |  |
| 25 – 34 | Reference | - |  |
| 35 – 44 | 2.05 (-0.39, 4.50) | 0.099 |  |
| 45 + | 4.55 (1.91, 7.20) | <0.001 |  |
| *BMI Category (kg/m^2^)* |  |  | 0.901 |
| Severely underweight (< 16) | -4.06 (-7.06, -1.07) | 0.008 |  |
| Underweight (16 – 18.49) | -3.99 (-6.08, -1.91) | <0.001 |  |
| Normal (18.5 – 24.99) | Reference | - |  |
| Overweight (> 25) | 1.68 (-1.91, 5.27) | 0.358 |  |
| *Country* |  |  | # |
| Ethiopia | 0.63 (-2.43, 3.69) | 0.686 |  |
| Georgia | -1.23 (-5.34, 2.88) | 0.558 |  |
| India | 5.24 (2.23, 8.26) | 0.001 |  |
| Moldova | 4.37 (1.21, 7.52) | 0.007 |  |
| Mongolia | Reference | - |  |
| South Africa | -7.68 (-10.64, -4.73) | <0.001 |  |
| Uganda | -4.64 (-7.90, -1.38) | 0.005 |  |
| *Smoking status* |  |  |  |
| Never smoked | * | * |  |
| Current smoker | * | * |  |
| Ex-smoker | * | * |  |
| *Number of cavities* |  |  |  |
| None | * | * |  |
| Single | * | * |  |
| Multiple | * | * |  |
