# Supplementary material for: Treatment outcomes in people with diabetes and multidrug-resistant tuberculosis (MDR TB) enrolled in the STREAM clinical trial
Source: PLOS Glob Public Health. 2025 Apr 1;5(4):e0004259. doi: 10.1371/journal.pgph.0004259 (PMC11960897; doi:10.1371/journal.pgph.0004259)
Supplement: S3 Table — (DOCX) [file pgph.0004259.s005.docx]

**S3 Table** Final piecewise longitudinal model for glucose levels (DM group)

|  | **Parameter estimate (95% CI)** | **P-value** | **P-value for interaction** |
| --- | --- | --- | --- |
| *Intercept* | 145.24 (49.97, 240.50) | 0.003 | # |
| *Baseline glucose* | 0.20 (0.06. 0.33) | 0.004 | # |
| *Time (Up to 20 weeks)* | -1.89 (-3.44, -0.34) | 0.017 | # |
| *Time (Slope difference from 20-76 weeks)* | 2.43 (0.52, 4.32) | 0.012 | # |
| *Treatment regimen* |  |  | 0.055 |
| Long | -56.29 (-134.43, 21.86) | 0.158 |  |
| Short | Reference | - |  |
| Oral | -29.10 (-68.73, 10.52) | 0.150 |  |
| Six-month | -26.44 (-68.23, 15.35) | 0.215 |  |
| *Female* | 2.57 (-36.28, 41.41) | 0.897 | 0.297 |
| *Age (years)* |  |  | 0.114 |
| 25 – 34 | Reference | - |  |
| 35 – 44 | 79.97 (16.71, 143.24) | 0.013 |  |
| 45 + | 58.66 (-0.23, 117.56) | 0.051 |  |
| *BMI Category (kg/m^2^)* |  |  | 0.209 |
| Severely underweight (< 16) | 55.17 (-60.02, 170.35) | 0.348 |  |
| Underweight (16 – 18.49) | 55.67 (8.95, 102.39) | 0.020 |  |
| Normal (18.5 – 24.99) | Reference | - |  |
| Overweight (> 25) | 24.83 (-18.31, 67.97) | 0.215 |  |
| *Country* |  |  | # |
| Georgia | 16.44 (-69.30, 102.17) | 0.707 |  |
| India | -40.64 (-84.47, 3.19) | 0.069 |  |
| Mongolia | Reference | - |  |
| *Smoking status* |  |  |  |
| Never smoked | * | * |  |
| Current smoker | * | * |  |
| Ex-smoker | * | * |  |
| *Number of cavities* |  |  |  |
| None | * | * |  |
| Single | * | * |  |
| Multiple | * | * |  |
