# Supplementary material for: Treatment outcomes in people with diabetes and multidrug-resistant tuberculosis (MDR TB) enrolled in the STREAM clinical trial
Source: PLOS Glob Public Health. 2025 Apr 1;5(4):e0004259. doi: 10.1371/journal.pgph.0004259 (PMC11960897; doi:10.1371/journal.pgph.0004259)
Supplement: S4 Table — (DOCX) [file pgph.0004259.s007.docx]

**S4 Table** Serious adverse events profiles

|  | **Non-DM**  **N=308 events** | **DM**  **N=63 events** |
| --- | --- | --- |
| ***SAEs, no. of events (% total events)*** | | |
| Ear and labyrinth | 35 (11.4) | 9 (14.3) |
| Respiratory, thoracic and mediastinal | 34 (11.0) | 5 (7.9) |
| Infections and infestations | 26 (8.4) | 4 (6.4) |
| Hepatobiliary | 21 (6.8) | 6 (9.5) |
| Investigations | 22 (7.1) | 4 (6.4) |
| Gastrointestinal | 19 (6.2) | 5 (7.9) |
| Metabolism and nutrition | 17 (5.5) | 4 (6.4) |
| Psychiatric | 21 (6.8) | 0 (0) |
| General | 18 (5.8) | 2 (3.2) |
| Surgical and medical procedures | 8 (2.6) | 8 (12.7) |
| Renal and urinary | 10 (3.3) | 3 (4.8) |
| Injury, poisoning and procedural complications | 11 (3.6) | 1 (1.6) |
| Social circumstances | 12 (3.9) | 0 (0) |
| Blood and lymphatic system | 10 (3.3) | 1 (1.6) |
| Cardiac disorders | 10 (3.3) | 1 (1.6) |
| Nervous system disorders | 7 (2.3) | 3 (4.8) |
| Neoplasms benign, malignant, and unspecified | 7 (2.3) | 1 (1.6) |
| Endocrine | 1 (0.3) | 5 (7.9) |
| Pregnancy, puerperium, and perinatal | 6 (2.0) | 0 (0) |
| Vascular | 5 (1.6) | 1 (1.6) |
| Musculoskeletal and connective tissue | 2 (0.7) | 1 (1.6) |
| Reproductive system and breast | 2 (0.7) | 0 (0) |
| Skin and subcutaneous tissue | 2 (0.7) | 0 (0) |
| Congenital, familial, and genetic | 1 (0.3) | 0 (0) |
| Eye | 1 (0.3) | 0 (0) |

|  | **Non-DM**  **N=889** | **DM**  **N=87** | **P-value** |
| --- | --- | --- | --- |
| ***SAEs, no. participants with an SAE***  ***(% total participants)*** | 193 (22) | 36 (41) | <0.001 |
| *Long regimen* | 47/147 (32) | 5/16 (31) | 0.953 |
| *Short regimen* | 95/421 (23) | 18/41 (44) | 0.002 |
| *Oral regimen* | 29/193 (15) | 5/17 (29) | 0.123 |
| *Six-month regimen* | 22/128 (17) | 8/13 (62) | <0.001 |
