# Supplementary material for: Treatment outcomes in people with diabetes and multidrug-resistant tuberculosis (MDR TB) enrolled in the STREAM clinical trial
Source: PLOS Glob Public Health. 2025 Apr 1;5(4):e0004259. doi: 10.1371/journal.pgph.0004259 (PMC11960897; doi:10.1371/journal.pgph.0004259)
Supplement: Attachment — Submitted filename: Response to Reviewers.docx [file pgph.0004259.s009.docx]

**Treatment outcomes in people with diabetes and multidrug-resistant tuberculosis (MDR TB) enrolled in the STREAM clinical trial.**

**Review Comments to the Author**
Reviewer #1: In this manuscript, the authors present a secondary analysis to determine the impact of comorbid diabetes on MDR-TB treatment outcomes. Like many to large cohorts, they attempt to draw power from a minority exposure within a large data set. In their evaluation, they did not identify significant differences in treatment outcomes, however they report non-significant outcomes as suggestive of differences being present, which is not consistent with the underlying data. While the finding that the absence of a significant difference between study groups may warrant presentation, the manner in which is represented is a bit misleading. Overall, there are challenges in both the description of the exposure and the outcome as outlined below, which limits the extent to which these data as presented contribute to the literature.

Major Comments:
Non-significant findings should be presented as not being different, rather than representing differences that were not significant.

We note the reviewer’s concern and have made edits to this effect in the Abstract, Results, and Discussion sections of the manuscript. All findings, except for the SAEs, have now been presented as comparable and not being different in all these sections.

The study would be improved by evaluation of their exposure of interest using glycosylated haemoglobin, rather than single random glucose measurements, as that may more accurately reflect the distribution of diabetes within this population.

We agree with the reviewer that the single random glucose measurement-based determination of exposure is a limitation of this study. Unfortunately, we do not have HbA1c values for this cohort of participants and are therefore reliant on surrogate markers such as baseline random glucose value (supplemented by information on concomitant medication in Stage 2 participants) to determine exposure status. We have now included this as a limitation in our Discussion section.

The authors note that they included change in treatment regimen as an unfavorable outcome, which is extremely common during MDR-TB treatment in order to optimally manage toxicities. While this is commonly represented in studies of DS-TB, often indicating emerging resistance, if the authors were to present this as a primary outcome, it should be presented in parallel with an analysis of unfavorable MDR-TB outcomes that exclude change in regimen.
The definition of favourable outcome used is the primary endpoint of the trial, and as such we do not wish to make changes to its definition. We acknowledge that this is a composite endpoint that includes TB outcomes, toxicity, lost to follow-up and agree it is useful to consider a more clinical, TB-related outcome, ignoring treatment changes. This is what the trial secondary endpoint of failure or recurrence (FoR) outcome does. We therefore consider the favourable outcome and FoR outcome together to cover the points the reviewer raises.

Finally, the authors fail to account for the dysglycemia of chronic illness commonly associated with TB disease, much of which self-resolves with immune recovery following TB treatment. As such, the presence of for example, an A1c of 6.6% resolving to 6.3%, might represent someone who is not truly diabetic beyond this temporary dysglycemia, and the analysis and discussion presented in the manuscript would need to take this into account.

Once again, we note the reviewer’s point around this and have made edits to the Discussion section to reflect on this aspect and to note limitations in our findings and conclusions, taking this phenomenon into consideration.

Reviewer #2: The review is well written.
I found no issue related to the statistical analyses performed in the study. The ethical considerations have been presented. The tables are well presented, and the numbers are correct. The conclusion is in line with the findings of the study.

We thank this reviewer for their assessment of our manuscript.

Reviewer #3:

1. The Abstract looks too long; can it be somewhat tightened?

We have now tightened the abstract and reduced it to 222 (from 313) words.

2. The statistical analyses involved well known techniques, such as Fisher's exact test, Chi-squared test (for proportions), and 2-sample z-tests/t-tests. It also involves fitting proportional hazards regression with time-to-event endpoints. If I am not mistaken, I do not see the assessment of the proportional hazards’ assumptions, which can be easily done using standard software, such as SAS, R, STATA, etc.

The proportional-hazards assumption was tested on the basis of Schoenfeld residuals. This was omitted in error from the methods and has been added to the statistical methods section (see in **bold** below).

*“Time-to-event endpoints (FoR and culture conversion) were analysed using Cox proportional hazard regression models (stratified by trial stage and regimen, if appropriate) to estimate hazard ratio between the DM and non-DM groups). All baseline factors considered potentially related to outcome as well as a-priori variables were included in a multivariable model and the best model selected using Akaike Information Criterion (AIC).* ***The proportional-hazards assumption was tested using Schoenfeld residuals and found to hold for all analyses****.”*

3. A piecewise longitudinal model of glucose control over time was fitted. This fitting was not mentioned in the statistical analysis section. Furthermore, the justifications behind the piecewise construction is not provided clearly.

Although we described fitting longitudinal models of glucose in the statistical methods, we had not specifically mentioned a piecewise exponential model. We have clarified this in the statistical methods section (see in **bold** below).

*“In addition, longitudinal glucose measures were summarized by visit and DM status and modelled using a linear mixed model by DM status, as glycaemic control appeared to be different in the two groups. Multiple functional forms for time were considered.* ***The best fitting model was a piecewise exponential model with knot at 20 weeks; 20 weeks was chosen as this was the best fit based on a simple plot of mean glucose level over time and made clinical sense since this coincided with the end of intensive phase of treatment****. Tests of interaction between demographic and baseline clinical characteristics with time were conducted to determine variables associated with glucose control.”*
